# Adult obesity diagnostic tool: A narrative review

**DOI:** 10.1097/MD.0000000000037946

**Published:** 2024-04-26

**Authors:** Xiaolong Liu, Mengxiao He, Yi Li

**Affiliations:** a School of Life & Environmental Sciences, Guilin University of Electronic Technology, Guilin, Guangxi, China; b School of Electronic Engineering and Automation, Guilin University of Electronic Technology, Guilin, Guangxi, China; c Rehabilitation College, Guilin Life and Health Career Technical College, Guilin, Guangxi, China; d School of Physical Education and Health, Guilin University, Guilin, Guangxi, China.

**Keywords:** adult obesity, evaluation indicators, measurement methods, narrative review

## Abstract

Obesity is a complex chronic metabolic disorder characterized by abnormalities in lipid metabolism. Obesity is not only associated with various chronic diseases but also has negative effects on physiological functions such as the cardiovascular, endocrine and immune systems. As a global health problem, the incidence and prevalence of obesity have increased significantly in recent years. Therefore, understanding assessment methods and measurement indicators for obesity is critical for early screening and effective disease control. Current methods for measuring obesity in adult include density calculation, anthropometric measurements, bioelectrical impedance analysis, dual-energy X-ray absorptiometry, computerized imaging, etc. Measurement indicators mainly include weight, hip circumference, waist circumference, neck circumference, skinfold thickness, etc. This paper provides a comprehensive review of the literature to date, summarizes and analyzes various assessment methods and measurement indicators for adult obesity, and provides insights and guidance for the innovation of obesity assessment indicators.

## 1. Introduction

Obesity refers to a state of disordered energy metabolism in the body caused by complex interactions between genetic and environmental factors. Prolonged periods of being in a relatively obese state not only affect an individual’s health and quality of life, but also lead to adverse changes in the structure and function of various body systems. It can even pose potential risks to an individual’s psychological well-being, resulting in negative effects such as anxiety, low self-esteem, depression, and more.^[1]^ According to the World Obesity Federation,^[2]^ By 2020, the global number of overweight or obese people will be approximately 2603 million, with a prevalence rate of 38%. In addition, the global number of obese people will be approximately 988 million, with an obesity rate of 14%. It is projected that by 2035, more than 4 billion people worldwide will be overweight or obese, representing more than half of the world’s population. Obesity not only affects the quality of life and life expectancy of individuals, but also places a significant economic burden on global healthcare systems.^[3]^ It is therefore essential to fully understand the diagnostic criteria and assessment indicators for obesity in order to provide support and new strategies for early intervention or prevention.

The body mass index (BMI) formula, originally proposed by Quetelet, was once considered an effective tool for screening for obesity. Due to its convenience, safety and low cost, this screening method was widely accepted and applied by the general public.^[4]^ However, after long-term large-scale application, it was found that this obesity screening tool has limited accuracy and is not applicable to all ethnic groups. Inaccurate and inaccurate diagnoses have a significant impact on the healthcare system. As a result, researchers have continuously explored new ways to diagnose obesity.^[5]^ Despite the may limitations of using BMI to diagnose obesity, it is undeniable that this indicator remains highly effective as a crude screening tool for large sample sizes. Long-term application of research suggests that the higher an individual’s BMI, the greater the need to perform additional assessments for potential metabolic abnormalities and complications.^[6]^ Despite the many limitations in using BMI for diagnosing obesity, additional information is required in practical diagnosis, such as details about fat distribution, waist circumference, subcutaneous fat thickness, and the impact of fat accumulation on individuals. However, it is undeniable that this indicator remains highly effective as a rough screening tool for large sample sizes. Additionally, the higher the BMI value, the more necessary it is to conduct additional assessments for potential metabolic abnormalities and complications. Similar to BMI, there are numerous methods for assessing obesity, but they generally have many limitations. Therefore, in practical diagnosis, it is necessary to obtain additional information such as fat distribution, waist circumference, subcutaneous fat thickness, and the effect of fat accumulation on individuals for a more accurate assessment of body adiposity. Subsequent research has continuously introduced various obesity assessment methods, mainly including density calculation, anthropometric measurements, bioelectrical impedance analysis, dual-energy X-ray absorptiometry, computer imaging, etc. The measured indicators mainly include weight, hip circumference, waist circumference, neck circumference, skinfold thickness and more.^[7]^ There are numerous indicators and evaluation methods for measuring obesity, each with its advantages and disadvantages, and different evaluation methods also have their unique applicability.^[8]^

This article reviews and analyzes the existing literature, consolidating and analyzing various methods of assessing adult obesity and measured indicators. This article reviews relevant research and application advances with the goal of providing theoretical references and insights for accurate screening and diagnostic technological innovations in obesity, early intervention in obesity, and innovative theories in obesity prevention.

## 2. Methods

This narrative review was performed by collecting clinical trials, basic research, and reviews on obesity diagnostic methods. Articles published in peer-reviewed scientific journals were included. Articles were excluded if they were not written in English or published in peer-reviewed scientific journals.

### 2.1. Search strategy

Literature searches were performed in PubMed and Web of Science databases from the date of database inception through January 2024. The search included the following keywords: adult obesity diagnosis, adult obesity measurement, adult obesity assessment, obesity diagnosis, obesity measurement, obesity assessment, obesity diagnostic methods, obesity measurement methods, obesity assessment methods, obesity measurement index, obesity assessment index.

### 2.2. Study selection

We searched for relevant articles and selected articles for further reading based on the title and abstract, all included articles were carefully discussed in the present review. One thousand, one hundred twenty-eight articles were included from the initial search and 77 articles were finally selected and discussed in the review. This narrative review does not require ethical approval, because no human/patient data were used.

## 3. Density measurement method

The density measurement method treats the human body as a physical entity composed of fat and nonfat tissues. By measuring the mass and volume values of the body and substituting them into a formula, this method calculates the body density and percentage of body fat.^[9]^ Volume measurement can be divided into the displacement method and the air displacement method. After obtaining volume data, these values are substituted into the density calculation formula to determine the percentage of body fat. The method of calculating human body density can be traced back to 1964, when Suzuki proposed the Suzuki equation for calculating human body density. In 1978,^[10]^ Jackson improved the algorithm and subsequently introduced the Jackson-Pollock equation.^[11]^ Both of these algorithms demonstrate high accuracy. The calculation methods for body fat percentage have developed to approximately a hundred different approaches, with some of the mainstream algorithms including the Siri equation^[12]^ and the Brozek equation,^[13]^ and others. The density measurement method can roughly estimate the total body fat and nonfat tissue content. However, its drawbacks include complexity in calculation, time-consuming procedures, and the inability to assess fat distribution. Additionally, for adolescent and elderly populations, the different tissue density compared to adults may lead to less accurate measurement results.

Due to its better match with the density estimation required by underwater weighing, the Siri equation is suggested to have relatively higher accuracy in situations where underwater weighing is used to determine body density. However, a drawback of the Siri equation is its relatively complex calculation process and computational challenges. Therefore, its use may be limited in scenarios such as large-scale epidemiological surveys, screening with large sample sizes, and situations where there are time constraints on detection.^[14]^ In addition, while this calculation method works well in certain populations and contexts, it is not universally applicable to individuals of all body types and constitutions. The simplicity of the Brozek equation makes it more feasible in practical applications, which is critical for large-scale studies and epidemiological investigations. Because of its simplicity, the Brozek equation was been widely used in early studies of body fat percentage, providing convenience for the collection and comparison of large amounts of data. However, the simplified structure of this calculation method sacrifices some accuracy, especially in situations with significant individual differences and complex body types, making it less suitable for studies that require greater accuracy in body fat percentage calculation.^[15]^ In addition, the introduction of age as a factor introduces variation in the applicability of the formula to different age groups, which could potentially affect the accuracy of the research results.

### 3.1. Air displacement plethysmography

Air displacement plethysmography is a method of estimating human body density based on the relationship between pressure and volume. During the measurement, the device measures the volume of air in the device chamber, subtracts the volume of air in the chamber after the person sits in it, and thus obtains the body volume value. Each measurement takes about 5 to 8 minutes.^[16]^ This method is based on the changes in volume of the subject inside the chamber and the law of conservation of mass. It derives the body fat content by accurately measuring the air volume and the weight of the subject. This method sets the densities of lean body tissue and fat tissue as constants, approximately 1.100 g/cm³ and 0.900 g/cm³, respectively.^[17]^ These concepts are based on the assumption that the density of human body tissues remains constant, and are therefore somewhat idealized. In practical applications, it is possible to introduce certain correction factors or combine them with other measurement methods to improve the accuracy of the calculated results. Representative devices for air displacement plethysmography include commercially available BOD POD chambers, PEA POD chambers, and others. The use of such devices typically results in body fat percentage errors in the range of 1% to 2%, showing high repeatability compared to other measurement methods and providing relatively accurate measurement results.^[18]^ Disadvantages include complexity of operation, high cost of equipment, and longer measurement time.^[19]^ Some studies suggest that air displacement plethysmography can accurately estimate and control body fat within 5% of reference measurements.^[20]^

### 3.2. Underwater weighing

Underwater weighing calculates body density by measuring the difference between the body on land and the body submerged in water, thereby estimating the content of adipose and non-adipose tissues. This method was first proposed by AKERS in 1969 and was once considered the “gold standard” because of its perceived relatively high efficacy and reliability.^[21]^ Participants are fully submerged in water while breathing through a tube connected to the outside environment. After volume and underwater weight are measured, body density and body fat percentage are calculated. Due to the higher density of human bone and muscle compared to water, individuals with greater lean mass weigh more in water, resulting in a lower body fat percentage.

Considering the presence of a certain amount of gas in human lungs, it is possible to estimate body fat percentage by assuming a model in which fat mass and fat-free mass have different densities. On the other hand, the volume of gas in the gastrointestinal tract varies from person to person (typically 100–150 mL in normal adults), and the values cannot be measured accurately. Therefore, there is a certain degree of error in the body density values obtained through by method.^[22]^ To accurately calculate body density using hydrostatic weighing, corrections should be made to the body volume based on the volume of air in the lungs and gastrointestinal tract during the measurement. Some studies demonstrate that when based on the 4-compartment model, the standard error of body fat percentage calculated using the underwater weighing method is between 1.3% and 1.5%.^[23]^ Nevertheless, underwater weighing remains the most classic method for estimating body composition. The principle is simple, and the error is relatively small compared to other methods of measurement. Disadvantages include higher facility and equipment requirements, longer measurement time, complicated procedures, significant influence of pulmonary and gastrointestinal gas volumes, and significant computational requirements. In addition, it is not suitable for individuals who cannot use a breathing apparatus underwater, who cannot fully submerge their bodies in water, or who cannot tolerate immersion in water.

## 4. Anthropometry

### 4.1. Body mass index

BMI is an indicator based on numerical values of body weight and height that assesses overweight or obesity in individuals. The calculation involves dividing the weight by the square of the height. The World Health Organization (WHO) previously defined individuals with a BMI ≥ 30 kg/m^2^ as obese and further categorized levels of obesity as follows: BMI ≥ 40.0 kg/m^2^ is classified as severe obesity (class 3 obesity); BMI between 35.0 kg/m^2^ and 39.9 kg/m^2^ is classified as moderate obesity (class 2 obesity); BMI between 30.0 kg/m^2^ and 39.9 kg/m^2^ is classified as mild obesity (class 1 obesity).^[24]^ The International Obesity Task Force has previously proposed that a BMI ≥ 25 kg/m^2^ be considered indicative of obesity.^[25]^ Researchers, in the long-term use of this method for diagnosing obesity and screening overweight or obese patients, have found that using BMI to assess obesity has the advantages of simplicity and wide applicability. However, its disadvantage is its inability to differentiate between fat and nonfat tissue content. In addition, it cannot accurately assess for edematous patients, those with malnutrition syndrome, those with age-related muscle loss, those with low body fat, and those with higher muscle content.^[26]^ When BMI is used to screen for obesity, it shows the characteristic of low sensitivity, and results are inconsistent when BMI is used to assess the linear relationship between obesity and various diseases.^[27]^ The main reason that BMI cannot be used as the sole diagnostic indicator of obesity is that the standards for assessing obesity using BMI and body fat percentage are inconsistent, with body fat percentage having stricter cutoffs and BMI having looser cutoffs. However, it is undeniable that in large sample studies, BMI holds a high diagnostic value for obesity. In addition, when BMI is combined with indicators such as waist circumference, hip circumference, body fat percentage, and others, the diagnosis of obesity becomes more accurate. Moreover, and more importantly, BMI, body fat percentage, waist-to-height ratio and other indicators can effectively predict the risk of hypertension and arterial sclerosis diseases.^[28]^

### 4.2. Waist circumference

Waist circumference (WC) refers to the horizontal circumference of the waist at the level of the navel. It is the most commonly used measurement index in clinical settings to assess central obesity. It can indirectly reflect the degree and distribution of fat accumulation in the waist and abdomen of the body.^[29]^ Obesity caused by abnormal accumulation of fat around the waist and abdomen is called central obesity or abdominal obesity. Excessive accumulation of fat around the waist and abdomen is closely associated with an increase in visceral fat mass. Previous research has consistently shown a strong correlation between the degree of central obesity and the risk of developing cardiovascular diseases, metabolic disorders, cancer, and other disease.^[30]^ Therefore, the discrimination of central obesity holds greater pathological significance. The WHO advocates the use of waist-to-hip ratio and WC to assess central obesity. These 2 indicators have simple measurements and wide applicability, and hold significant importance for primary screening of obesity.^[31]^ According to the WHO, a diagnosis of abdominal obesity is made when the WC is equal to or >102 cm for men and 88 cm for women. The International Diabetes Federation suggests that a diagnosis of abdominal obesity is established when the WC is equal to or >90 cm for men and 80 cm for women.^[32]^ The 3 evaluation standards have significant differences, and their screening accuracy varies widely. Therefore, it is particularly important to tailor diagnostic criteria based on factors such as economic conditions, racial and gender differences, and other contextual considerations in different countries.

Measurement methods for WC include: (1) The circumference at the horizontal plane where the highest point of the iliac crest is located, as used by the National Institutes of Health in the United States,^[33]^ (2) Suggested by the InterASIA International Collaboration on Cardiovascular Disease, the measurement is the circumference in the horizontal plane 1 centimeter above the navel,^[34]^ (3) The circumference in the horizontal plane of the midpoint between the iliac crest and the lower edge of the rib cage at the midaxillary line, as proposed by the WHO.^[35]^ As one of the evaluation indicators for metabolic syndrome, WC has characteristics such as easy measurement and low cost. It can effectively estimate the risk of developing cardiovascular disease and diabetes.^[36]^ Research shows that using WC to assess central obesity is more sensitive and accurate than BMI and waist-to-hip ratio.^[37]^ Due to the neglect of the influence of height, relying solely on WC for obesity diagnosis exhibits a limitation in accuracy. This is particularly evident in special populations such as those with exceptionally tall or short stature, individuals with excessive food intake, those with high abdominal muscle content, and pregnant women.^[38]^

### 4.3. Hip circumference

Hip circumference (HC) refers to the circumference of the most prominent point of the pelvis around the buttocks. Early studies suggested using an HC >101.5 cm as an adjunct diagnostic criterion for obesity in women. If the HC exceeds 101.5 cm in women, a diagnosis of obesity can be directly made if her BMI is greater than or equal to 28 kg/m^2^.^[39]^ In addition, some studies have shown a positive correlation between HC and the risk of polycystic ovary syndrome in women.^[40]^ Conversely, some researchers have suggested that a larger HC can effectively reduce the risk of developing metabolic syndrome.^[41]^ There is increasing evidence that a reduction in HC is associated with an increased risk of cardiovascular disease, coronary heart disease, diabetes and premature death in women.^[42]^ It can be confirmed that when HC is used in conjunction with BMI as a primary screening method for obesity, the assessment of individuals with a higher content of hip skeletal muscle may be less accurate. However, due to the simplicity of measuring a single value and the relatively uniform measurement method, it is more economical and convenient compared to the measurement of other indicators.

### 4.4. Body adiposity index

The body adiposity index (BAI) is a method for assessing obesity based on height and HC. The specific calculation method is as follows: BAI = HC (cm)/Height (m)^1.5–18^, the BAI can effectively reflect the total body fat content. It was first proposed by Bergman et al and has been successfully applied to Mexican American and African American populations using this calculation method. The BAI can provide a comprehensive reflection of total body fat content. It was originally proposed by Bergman et al and has previously been used to assess body adiposity in Mexican American and African American populations.^[43]^ Some studies suggest that the BAI has a significant correlation with indicators such as blood uric acid levels, blood triglyceride levels, and fasting blood glucose concentration. It also shows highly significant positive correlations with WC, BMI, waist-to-hip ratio, and other obesity assessment indices.^[44]^ Due to the simple measurement of height and HC, this method has advantages such as simplicity and ease of use. Although the BAI is highly correlated with dual-energy X-ray scans, its value in predicting obesity is lower than WC and BMI, but higher than waist-to-hip ratio.^[45]^ In addition, some studies have confirmed that the BAI may not accurately assess the degree of obesity in individuals with body fat below 15%, and its accuracy is limited when applied to populations with extremely low or high body fat.^[46]^

### 4.5. Waist-to-hip ratio

Waist-to-hip ratio (WHR) refers to the ratio of WC to HC, and this ratio was once thought to be associated with the risk of developing cardiovascular disease in the human body.^[47]^ The WHR, originally proposed by the WHO as an extrinsic measure to determine abdominal obesity, has significant variations in assessment standards worldwide due to differences in WC measurement methods. According to WHO guidelines, a WHR greater than or equal to 0.90 for men and greater than or equal to 0.85 for women is considered central obesity.^[48]^ WHR, which requires the measurement of 2 values, is relatively cumbersome compared to single-site measurements. It has certain limitations in assessing central obesity, as individuals with the same WHR do not necessarily have the same amount of waist and abdominal fat.^[49]^ WHR has a higher correlation with abnormalities in blood lipids, uric acid, blood glucose, blood pressure, and other indicators, especially in the assessment of type 2 diabetes and lipid abnormalities, where WHR accuracy is significantly higher than BMI.^[50]^ Previous studies have confirmed a significant correlation between myocardial infarction and an elevated WHR.^[51]^ Some studies also suggest that WHR serves as a moderate screening marker for moderate to severe obstructive sleep apnea and is an independent risk factor for obstructive sleep apnea.^[52]^

### 4.6. Waist-to-height ratio

Waist-to-height ratio (WHtR) is also known as WHtR, waist-to-height ratio, and other terms. Its calculation method is as follows: WHtR = WC/Height. International standards often consider a WHtR >0.5 as a diagnostic criterion for central obesity.^[53]^ The characteristic of WHtR is its large correlation coefficient with WC and virtually no gender differences, making it an ideal screening tool for identifying obese individuals. In addition, WHtR is also used to predict diseases such as coronary atherosclerosis, heart disease, metabolic syndrome, and adult-onset diabetes, and serves as a tool to assess the risk of developing these diseases in different individuals.^[54]^ Previous studies have suggested that the WHtR has a better correlation with many cardiovascular and metabolic components compared to other adiposity indices. An elevated WHtR is more strongly associated with hypertension, hypercholesterolemia, and elevated low-density lipoprotein cholesterol. It demonstrates practical utility in large-scale assessments of body adiposity and effectively enhances the ability to identify cardiovascular metabolic risk.^[55]^ Some studies also suggest that WHtR has significantly higher accuracy in predicting or screening for lipid abnormalities compared to BMI, WHR, WC, and other measures.^[56]^

### 4.7. Neck circumference

Neck circumference refers to the horizontal circumference at the narrowest point below the Adam apple. As one of the indicators of central obesity, neck circumference may indirectly reflect the subcutaneous and visceral fat content of the upper body.^[57]^ Neck circumference is also a good predictor of the risk of type 2 diabetes, cardiovascular disease, metabolic syndrome, and is highly correlated with the risk of developing obstructive sleep apnea and hypoventilation syndrome.^[58]^ An earlier cross-sectional study of 376 participants found that neck circumference is effective in identifying excess thyroid fat in the body, thereby determining central fat content, and the accuracy is satisfactory.^[59]^ Another cohort study of 1421 participants found a positive correlation between changes in neck circumference and visceral fat area. This suggests that using neck circumference as a diagnostic tool for abdominal obesity is practical and effective.^[60]^ In addition, neck circumference is considered a promising tool for identifying individuals susceptible to and at high risk for cardiovascular disease.^[61]^ Previous studies have shown that when the neck circumference is greater than or equal to 37.00 cm for men and greater than or equal to 34.00 cm for women, additional assessment of obesity is required to diagnose the presence of obesity.^[62]^ It is worth noting that when neck circumference is used to assess obesity in different racial/ethnic groups, there are significant differences in standards. Therefore, it is advisable to implement diagnostic criteria tailored to the different ethnicities within each country. Neck circumference has several advantages in practical application, such as simplicity of measurement and high accuracy, stability and repeatability of measurement results.^[63]^ Neck circumference can be used as an alternative indicator to BMI and WC for assessing central obesity.^[64]^ One drawback, however, is that neck circumference standards vary widely among different ethnic groups, resulting in significant differences in criteria.

### 4.8. Skinfold thickness

Various instruments can be used to measure local skinfold thickness values, which can then be used to estimate body density and body fat percentage. Skinfold thickness measurement methods mainly include caliper measurement, X-ray measurement, ultrasonic measurement, etc.^[65]^ The main measurement areas include triceps skinfold, calf skinfold, subscapular skinfold, biceps skinfold, abdomen skinfold, suprailiac skinfold, quadriceps skinfold, etc. Two to seven sites are selected each time for repeated measurements and the results are averaged.^[66]^ Skinfold thickness methods are easy to measure, with a variety of tools available, cost effective, and suitable for large scale measurements. However, the drawback is that the accuracy of the measurements is lower compared to other methods. Factors such as the force applied by the tester when pinching the skinfold, the duration of caliper compression at the measurement site, the subject’s skin laxity, skin thickness, and the presence of edema can all affect test results.

### 4.9. Abdominal body shape index

The abdominal body shape index (ABSI) is an indicator of central obesity that accurately reflects the distribution of abdominal fat throughout the body.^[67]^ The ABSI calculation method is:


$$\begin{array}{l} ABSI=\frac{WC}{\left(BMI^{2/3}\right)\times \left(Height^{1/2}\right)} \end{array}$$

ABSI is highly correlated with the risk of chronic diseases such as central obesity, cardiovascular disease, and metabolic syndrome, and compared to traditional BMI, it can more accurately assess the degree of central obesity in the body. At the same time, ABSI has higher accuracy in identifying metabolic diseases such as cardiovascular disease, providing more accurate information for large-scale cardiovascular health risk assessments.^[36]^ In addition, the ABSI calculation formula combines multiple biological parameters, taking into account factors such as abdominal fat, total weight and height, to provide a more comprehensive assessment of individual health risk. The disadvantage of ABSI is the complexity of data collection and the challenge of standardization. At the same time, the calculation of ABSI requires multiple biological parameters, including WC, BMI, and height, which can increase the complexity of data collection in practical clinical and research settings. Second, there is currently no established uniform and effective standardization system for ABSI. Different studies and clinical practices may use different calculation methods and standards, thereby reducing the consistency of comparison and application. Further research is needed to validate the applicability of the ABSI in different populations and to explore its predictive accuracy for health status in different populations. On the other hand, the combination of ABSI with biological markers such as inflammatory indicators and metabolites to enhance its comprehensiveness in multifactorial risk assessment is also one of the future research hotspots. Overall, the ABSI shows potential value in assessing central obesity and chronic disease risk, but its application requires further research and standardization. In the future, with a deeper understanding of the impact of central obesity and technological advances, the ABSI may become a more accurate and comprehensive tool for health assessment.

## 5. Bioelectrical impedance analysis

Bioelectrical impedance analysis (BIA) is a method that utilizes the different conductivity coefficients of adipose tissue and other tissues in the body. It calculates body impedance and then calculates body fat percentage based on the regression equation between body fat content and body impedance. Commonly used devices include two-electrode, four-electrode, six-electrode, and eight-electrode devices.^[68]^ The accuracy of BIA depends on factors such as humidity and tightness of contact between the subject and the electrodes, room temperature, the subject’s skin temperature of, the subject’s hydration level, the precision of the testing device, and the algorithm used.^[69]^ Body fat percentage, is the ratio of body fat mass to total body mass, which directly reflects the body fat content and ratio. Previous studies have proposed criteria for diagnosing obesity: a body fat percentage greater than or equal to 25% for men and greater than or equal to 35% for women.^[70]^ Subsequent studies have found that normal-weight individuals may have abnormal body fat content, especially in the case of normal-weight obesity. In this population, individuals are of normal weight but have a significant proportion of fat in their body composition. It is challenging to identify this group using external measurements such as BMI, WC, and HC. Therefore, using BIA to analyze body fat percentage becomes particularly important. BIA can comprehensively calculate the content of skeletal muscle and adipose tissue in the body, and the stability of measurements is relatively high within the same population at different times. However, its limitations include the influence of various factors on the measurement results, its unsuitability for measuring local fat content, and its applicability to epidemiologic surveys in different populations.^[71]^ In addition, due to the large number of impedance equations, it remains to be verified whether different calculation methods are applicable to populations in different countries.

## 6. Dual-energy X-ray absorptiometry

Dual-energy X-ray absorptiometry (DEXA) is a procedure that uses X-rays to measure bone density and tissue composition. This technique involves directing X-rays into the human body and analyzing the absorption characteristics of different tissues. It measures and analyzes the amount of bone, fat, and lean tissue in the body.^[72]^ DEXA can accurately assess body fat content and distribution, which is critical for evaluating central obesity and fat distribution in different regions of the body. DEXA collects X-ray absorption data from bones and tissues, producing high-resolution images that allow precise measurement of fat and lean body tissue at both the global and local levels. This precision gives DEXA a unique advantage in diagnosing obesity and providing insight into fat distribution patterns. Overall, DEXA has an extremely high correlation with computed tomography measurements, good repeatability, and excellent accuracy.^[73]^ Despite the high-precision advantages of DEXA, the issue of radiation exposure must be considered in practical applications. Therefore, when using DEXA, it is important to balance its benefits and risks. In addition, the disadvantages of DEXA include higher test costs compared to other methods, insufficient precision in assessing waist and abdominal fat content, and susceptibility to factors such as gender and age.^[74]^

## 7. Computer imaging method

Computer imaging methods mainly include magnetic resonance imaging (MRI) and computed tomography (CT). Both MRI and CT can accurately differentiate and measure visceral or subcutaneous fat, making them the gold standard for determining and measuring obesity. The advantage of CT is its extremely high accuracy and minimal factors that can affect the measurement results. However, this method is associated with longer measurement times, higher costs, and high equipment dependency.^[75]^ In addition, CT involves radiation and is therefore rarely used for obesity screening. Improving imaging quality while effectively reducing radiation dose is also one of the focus areas for future research. Compared to CT, MRI can noninvasively measure human spatial information and tissue structure. The advent of MRI has addressed the long-standing limitation of CT in capturing anatomical information within the body. MRI has a wide range of applications, but it has long acquisition times, high acquisition costs, and is not suitable for patients with metal implants.^[76]^

## 8. Limitations

There are some limitations to our research. First, this study did not include all relevant literature, and there may be missing reports of obesity diagnostic methods that are not included in the literature database or not widely reported. On the other hand, various obesity diagnostic methods that have been reported are proposed for different regions or ethnicities, which may limit the comparability of research results. In addition, the accuracy of various obesity diagnostic methods can be easily influenced by nonstandard measuring tools, nonstandard measuring postures, measuring environments, and nonstandard measuring operations, which may lead to biased research results. Therefore, the authors believe that more high-quality research on obesity diagnostic methods is needed.

## 9. Conclusion

There are numerous indicators and methods for measuring obesity, each with its own advantages and disadvantages. Different assessment methods also have unique applicability. When assessing obesity, differences in body composition due to factors such as gender, age, race and ethnicity should not be overlooked. In practice, selection should be based on actual conditions. The combination of multiple measures, the use of double standards, and the adoption of multi-criteria screening strategies are critical to increasing the accuracy of obesity assessment. With the development of biomedical, BIA with its safety, speed and accuracy shows promising potential in screening and assessment of obesity, demonstrating broad application prospects.

## Acknowledgments

This work was supported by The Research Basic Ability Enhancement Program for Young and Middle-aged Teachers of Guangxi (2023KY1989), Guangxi Graduate Education Innovation Program Project (YCSW2021109), and Natural Science Research Program of Guilin Life and Health Career Technology College (2023GKA002).

## Author contributions

**Conceptualization:** Xiaolong Liu.

**Data curation:** Mengxiao He, Yi Li.

**Investigation:** Yi Li.

**Supervision:** Yi Li.

**Writing – original draft:** Xiaolong Liu.

**Writing – review & editing:** Xiaolong Liu.

## References

[1] Masood B Moorthy M. Causes of obesity: a review. Clin Med (Lond). 2023;23:284–91. 10.7861/clinmed.2023-0168 PMC10541056 37524429

[2] World Obesity Federation. World Obesity Atlas 2023. 2023;10–1. https://www.worldobesityday.org/assets/downloads/World_Obesity_Atlas_2023_Report.pdf.

[3] Chew NWS Ng CH Tan DJH. The global burden of metabolic disease: Data from 2000 to 2019. Cell Metab. 2023;35:414–28.e3. 36889281 10.1016/j.cmet.2023.02.003

[4] Gutin I. In BMI we trust: reframing the Body Mass Index as a measure of health. Soc Theory Health. 2018;16:256–71. 31007613 10.1057/s41285-017-0055-0 PMC6469873

[5] Bray GA. Beyond BMI. Nutrients. 2023;15:2254. 37242136 10.3390/nu15102254 PMC10223432

[6] Khanna D Peltzer C Kahar P. Body Mass Index (BMI): a screening tool analysis. Cureus. 2022;14:e22119. 35308730 10.7759/cureus.22119 PMC8920809

[7] Nimptsch K Konigorski S Pischon T. Diagnosis of obesity and use of obesity biomarkers in science and clinical medicine. Metabolism. 2019;92:61–70. 30586573 10.1016/j.metabol.2018.12.006

[8] Blue MNM Tinsley GM Ryan ED. Validity of body-composition methods across racial and ethnic populations. Adv Nutr. 2021;12:1854–62. 33684215 10.1093/advances/nmab016 PMC8528114

[9] Kuriyan R. Body composition techniques. Indian J Med Res. 2018;148:648–58. 30666990 10.4103/ijmr.IJMR_1777_18 PMC6366261

[10] Nagamine S Suzuki S. Anthropometry and body composition of Japanese young men and women. Hum Biol. 1964;36:8–15. 14122590

[11] Jackson AS Pollock ML. Generalized equations for predicting body density of men. Br J Nutr. 1978;40:497–504. 718832 10.1079/bjn19780152

[12] Sarría A García-Llop LA Moreno LA. Skinfold thickness measurements are better predictors of body fat percentage than body mass index in male Spanish children and adolescents. Eur J Clin Nutr. 1998;52:573–6. 9725657 10.1038/sj.ejcn.1600606

[13] Brozek J Grande F Anderson JT. Densitometric analysis of body composition: revision of some quantitative assumptions. Ann N Y Acad Sci. 2010;110:113–40. 10.1111/j.1749-6632.1963.tb17079.x 14062375

[14] Fedewa MV Nickerson BS Tinsley GM. Examining race-related error in two-compartment models of body composition assessment: a systematic review and meta-analysis. J Clin Densitom. 2021;24:156–68. 31810770 10.1016/j.jocd.2019.10.002

[15] González-Arellanes R Urquidez-Romero R Rodríguez-Tadeo A. Agreement between laboratory methods and the 4-compartment model in assessing fat mass in obese older Hispanic-American adults. Clin Nutr. 2021;40:3592–600. 33419614 10.1016/j.clnu.2020.12.020

[16] Fields DA Gunatilake R Kalaitzoglou E. Air displacement plethysmography: cradle to grave. Nutr Clin Pract. 2015;30:219–26. 25761768 10.1177/0884533615572443

[17] Muntean P Popa A Miclos-Balica M. Learning effects in air displacement plethysmography. Life (Basel). 2023;13:1315. 37374098 10.3390/life13061315 PMC10304159

[18] Muntean P Micloș-Balica M Popa A. Reliability of repeated trials protocols for body composition assessment by air displacement plethysmography. Int J Environ Res Public Health. 2021;18:10693. 34682439 10.3390/ijerph182010693 PMC8535236

[19] Mazahery H Von Hurst PR McKinlay CJD. Air displacement plethysmography (pea pod) in full-term and preterm infants: a comprehensive review of accuracy, reproducibility, and practical challenges. Matern Health Neonatol Perinatol. 2018;4:12–22. 29951209 10.1186/s40748-018-0079-z PMC6011189

[20] Kuriyan R Thomas T Ashok S. A 4-compartment model based validation of air displacement plethysmography, dual energy X-ray absorptiometry, skinfold technique & bio-electrical impedance for measuring body fat in Indian adults. Indian J Med Res. 2014;139:700–7. 25027079 PMC4140034

[21] Golja P Robič Pikel T Zdešar Kotnik K. Direct comparison of (anthropometric) methods for the assessment of body composition. Ann Nutr Metab. 2020;76:183–92. 32640459 10.1159/000508514

[22] Heymsfield SB Ebbeling CB Zheng J. Multi-component molecular-level body composition reference methods: evolving concepts and future directions. Obes Rev. 2015;16:282–94. 25645009 10.1111/obr.12261 PMC4464774

[23] Nickerson BS Esco MR Bishop PA. Impact of measured vs. predicted residual lung volume on body fat percentage using underwater weighing and 4-Compartment Model. J Strength Cond Res. 2017;31:2519–27. 27806008 10.1519/JSC.0000000000001698

[24] WHO. Obesity. Preventing and Managing the Global Epidemic. Report of a WHO Consultation on Obesity. Geneva: WHO, 1998. 11234459

[25] WHO. The Asia-Pacific Perspective: Redefining Obesity and Its Treament. Geneva: WHO, 2000.

[26] Okorodudu DO Jumean MF Montori VM. Diagnostic performance of body mass index to identify obesity as defined by body adiposity: a systematic review and meta-analysis. Int J Obes (Lond). 2010;34:791–9. 20125098 10.1038/ijo.2010.5

[27] Romero-corral A Somers VK Sierra-johnson J. Accuracy of body mass index in diagnosing obesity in the adult general population. Int J Obes (Lond). 2008;32:959–66. 18283284 10.1038/ijo.2008.11 PMC2877506

[28] Oliveros E Somers VK Sochor O. The concept of normal weight obesity. Prog Cardiovasc Dis. 2014;56:426–33. 24438734 10.1016/j.pcad.2013.10.003

[29] Ross R Neeland IJ Yamashita S. Waist circumference as a vital sign in clinical practice: a Consensus Statement from the IAS and ICCR working group on visceral obesity. Nat Rev Endocrinol. 2020;16:177–89. 32020062 10.1038/s41574-019-0310-7 PMC7027970

[30] Campana EMG Brandão AA. Waist circumference: a parameter of vascular health. Arq Bras Cardiol. 2022;119:265–6. 35946688 10.36660/abc.20220508 PMC9363067

[31] Nishida C Ko GT Kumanyika S. Body fat distribution and noncommunicable diseases in populations: overview of the 2008 WHO expert consultation on Waist Circumference and Waist-Hip Ratio. Eur J Clin Nutr. 2010;64:2–5. 19935820 10.1038/ejcn.2009.139

[32] Bashir MA Yahaya AI Muhammad M. Prevalence of central obesity in Nigeria: a systematic review and meta-analysis. Public Health. 2022;206:87–93. 35436651 10.1016/j.puhe.2022.02.020

[33] Patry-Parisien J Shields M Bryan S. Comparison of waist circumference using the World Health Organization and National Institutes of Health protocols. Health Rep. 2012;23:53–60. 23061265

[34] Ross R Berentzen NT Bradshaw AJ. Does the relationship between waist circumference, morbidity and mortality depend on measurement protocol for waist circumference. Obes Rev. 2008;9:312–25. 17956544 10.1111/j.1467-789X.2007.00411.x

[35] Menke A Muntner P Wildman RP. Measure of adiposity and cardiovascular disease risk factors. Obesity (Silver Spring). 2007;15:785–95. 17372330 10.1038/oby.2007.593

[36] Jayedi A Soltani S Zargar MS. Central fatness and risk of all cause mortality: systematic review and dose–response meta-analysis of 72 prospective cohort studies. BMJ. 2020;23:m3324. 10.1136/bmj.m3324 PMC7509947 32967840

[37] Bosomworth NJ. Normal-weight central obesity: unique hazard of the toxic waist. Can Fam Physician. 2019;65:399–408. 31189627 PMC6738397

[38] Fang H Berg E Cheng X. How to best assess abdominal obesity. Curr Opin Clin Nutr Metab Care. 2018;21:360–5. 29916924 10.1097/MCO.0000000000000485 PMC6299450

[39] Zhou BF. Effect of body mass index on all-cause mortality and incidence of cardiovascular diseases-report for meta-analysis of prospective studies open optimal cut-off points of body mass index in Chinese adults. Biomed Environ Sci. 2002;15:245–52. 12500665

[40] Mawaddatina T Budihastuti UR Rahayu D. Waist circumference, hip circumference, arm span, and waist-to-hip ratio high risk of polycystic ovarian syndrome. Scott Med J. 2021;66:186–90. 34661497 10.1177/00369330211043206

[41] Parker ED Pereira MA Stevens J. Association of hip circumference with incident diabetes and coronary heart disease: the Atherosclerosis risk in communities study. Am J Epidemiol. 2009;169:837–47. 19224980 10.1093/aje/kwn395 PMC2727216

[42] Lanfer A Mehlig K Heitmann BL. Does change in hip circumference predict cardiovascular disease and overall mortality in Danish and Swedish women? Obesity (Silver Spring, Md.). 2014;22:957–63. 23963732 10.1002/oby.20604

[43] Tur JA Bibiloni MDM. Anthropometry, body composition and resting energy expenditure in human. Nutrients. 2019;11:1891. 31416130 10.3390/nu11081891 PMC6724063

[44] Zamaninour N Ansar H Pazouki A. Relationship between modified body adiposity index and a body shape index with biochemical parameters in bariatric surgery candidates. Obes Surg. 2020;30:901–9. 31898041 10.1007/s11695-019-04256-x

[45] Schulze MB Thorand B Fritsche A. Body adiposity index, body fat content and incidence of type 2 diabetes. Diabetologia. 2012;55:1660–7. 22349074 10.1007/s00125-012-2499-z

[46] Chang H Simonsick EM Ferrucci L. Validation study of the body adiposity index as a predictor of percent body fat in older individuals: findings from the BLSA. J Gerontol A Biol Sci Med Sci. 2014;69:1069–75. 24158764 10.1093/gerona/glt165 PMC4158412

[47] Myint PK Kwok CS Luben RN. Body fat percentage, body mass index and waist-to-hip ratio as predictors of mortality and cardiovascular disease. Heart. 2014;100:1613–9. 24966306 10.1136/heartjnl-2014-305816

[48] Burton RF. The waist-hip ratio: a flawed index. Ann Hum Biol. 2020;47:629–31. 32892641 10.1080/03014460.2020.1820079

[49] Haufs MG Zöllner YF. Waist-Hip Ratio more appropriate than Body Mass Index. Dtsch Arztebl Int. 2020;117:659. 10.3238/arztebl.2020.0659a PMC7829451 33357347

[50] Jayedi A Soltani S Motlagh SZ. Anthropometric and adiposity indicators and risk of type 2 diabetes: systematic review and dose-response meta-analysis of cohort studies. BMJ. 2022;18:e067516. 10.1136/bmj-2021-067516 PMC8764578 35042741

[51] Cao Q Yu S Xiong W. Waist-hip ratio as a predictor of myocardial infarction risk: a systematic review and meta-analysis. Medicine (Baltim). 2018;97:e11639. 10.1097/MD.0000000000011639 PMC6078643 30045310

[52] Wang Y Mao L Zhang X. Waist-hip ratio is an independent predictor of moderate-to-severe OSA in nonobese males: a cross-sectional study. BMC Pulm Med. 2022;22:151. 35459124 10.1186/s12890-022-01886-3 PMC9034636

[53] Bajpai A. Waist-to-height ratio-time for a new obesity metric? Indian J Pediatr. 2022;89:534–5. 35277810 10.1007/s12098-022-04173-5

[54] Tewari A Kumar G Maheshwari A. Comparative evaluation of waist-to-height ratio and BMI in predicting adverse cardiovascular outcome in people with diabetes: a systematic review. Cureus. 2023;15:e38801. 37303408 10.7759/cureus.38801 PMC10250251

[55] Peer N Lombard C Steyn K. Waist-to-height ratio is a useful indicator of cardio-metabolic risk in South Africa. Fam Pract. 2020;37:36–42. 31504474 10.1093/fampra/cmz044

[56] Zhong X Chang L Wenyan N. Identifying obesity indicators which best correlate with type 2 diabetes in a Chinese population. BMC Public Health. 2012;12:732–37. 22937748 10.1186/1471-2458-12-732 PMC3490952

[57] Pei X Liu L Imam MU. Neck circumference may be a valuable tool for screening individuals with obesity: findings from a young Chinese population and a meta-analysis. BMC Public Health. 2018;18:529–30. 29678132 10.1186/s12889-018-5448-z PMC5910608

[58] Luo Y Ma X Shen Y. Neck circumference as an effective measure for identifying cardio-metabolic syndrome: a comparison with waist circumference. Endocrine. 2017;55:822–30. 27796813 10.1007/s12020-016-1151-y

[59] Filgueiras MS Albuquerque FM Castro APP. Neck circumference cutoff points to identify excess android fat. J Pediatr (Rio J). 2020;96:356–63. 30731052 10.1016/j.jped.2018.11.009 PMC9432153

[60] Cao W Xu Y Shen Y. Change of neck circumference in relation to visceral fat area: a Chinese community-based longitudinal cohort study. Int J Obes (Lond). 2022;46:1633–7. 35672353 10.1038/s41366-022-01160-w PMC9395262

[61] Han JS Kim YH. Neck circumference and incidence of cerebrovascular disease over 12 years among Korean adults. Osong Public Health Res Perspect. 2022;13:71–9. 35255680 10.24171/j.phrp.2021.0277 PMC8907609

[62] Liu Y Zhang X Lee J. Genome-wide association study of neck circumference identifies sex-specific loci independent of generalized adiposity. Int J Obes (Lond). 2021;45:1532–41. 33907307 10.1038/s41366-021-00817-2 PMC8236408

[63] Padilha CM Pescuma JMS Rodrigues ALCC. Neck circumference as a marker of body adiposity in young to middle-aged adults. Nutrition. 2022;93:111496. 34678716 10.1016/j.nut.2021.111496

[64] Xu Y Li X Hu T. Neck circumference as a potential indicator of pre-sarcopenic obesity in a cohort of community-based individuals. Clin Nutr. 2024;43:11–7. 37992633 10.1016/j.clnu.2023.11.006

[65] Fosbøl MØ ZB. Contemporary methods of body composition measurement. Clin Physiol Funct Imaging. 2015;35:81–97. 24735332 10.1111/cpf.12152

[66] Forte GC Rodrigues CAS Mundstock E. Can skinfold thickness equations be substituted for bioimpedance analysis in children? J Pediatr (Rio J). 2021;97:75–9. 32084440 10.1016/j.jped.2019.12.006 PMC9432255

[67] Christakoudi S Tsilidis KK Muller DC. A Body Shape Index (ABSI) achieves better mortality risk stratification than alternative indices of abdominal obesity: results from a large European cohort. Sci Rep. 2020;10:14541. 32883969 10.1038/s41598-020-71302-5 PMC7471961

[68] Moonen HPFX Van Zanten ARH. Bioelectric impedance analysis for body composition measurement and other potential clinical applications in critical illness. Curr Opin Crit Care. 2021;27:344–53. 33967207 10.1097/MCC.0000000000000840 PMC8270506

[69] Ward LC. Bioelectrical impedance analysis for body composition assessment: reflections on accuracy, clinical utility, and standardisation. Eur J Clin Nutr. 2019;73:194–9. 30297760 10.1038/s41430-018-0335-3

[70] Lahav Y Epstein Y Kedem R. A novel body circumferences-based estimation of percentage body fat. Br J Nutr. 2018;119:720–5. 29553036 10.1017/S0007114518000223

[71] Elia M. Body composition by whole-body bioelectrical impedance and prediction of clinically relevant outcomes:overvalued or underused. Eur J Clin Nutr. 2013;67:S60–70. 23299873 10.1038/ejcn.2012.166

[72] Marra M Sammarco R De Lorenzo A. Assessment of body composition in health and disease using Bioelectrical Impedance Analysis (BIA) and Dual Energy X-Ray Absorptiometry (DXA): a critical overview. Contrast Media Mol Imaging. 2019;2019:3548284. 31275083 10.1155/2019/3548284 PMC6560329

[73] Lu YC Lin YC Yen AM. Dual-energy X-ray absorptiometry-assessed adipose tissues in metabolically unhealthy normal weight Asians. Sci Rep. 2019;9:17698. 31776349 10.1038/s41598-019-53557-9 PMC6881341

[74] Micklesfield LK Goedecke JH Punyanitya M. Dual-energy X-ray performs as well as clinical computed tomography for the measurement of visceral fat. Obesity (Silver Spring). 2012;20:1109–14. 22240726 10.1038/oby.2011.367 PMC3343346

[75] Xu JJ Boesen MR Hansen SL. Assessment of liver fat: dual-energy CT versus conventional CT with and without contrast. Diagnostics (Basel). 2022;12:708. 35328261 10.3390/diagnostics12030708 PMC8946969

[76] Ünal E Karaosmanoğlu AD Akata D. Invisible fat on CT: making it visible by MRI. Diagn Interv Radiol. 2016;22:133–40. 26782156 10.5152/dir.2015.15286 PMC4790064

